# Using mean dose rate to compare relative dosimetric efficiency with respect to source type and source change schedules for HDR brachytherapy°

**DOI:** 10.1120/jacmp.v14i6.4239

**Published:** 2013-11-04

**Authors:** Stephen D. Davis, William Parker, Michael D.C. Evans

**Affiliations:** ^1^ Department of Medical Physics McGill University Health Centre Montreal QC Canada; ^2^ Medical Physics Unit McGill University Montreal QC Canada

**Keywords:** brachytherapy, HDR, dosimetric efficiency

## Abstract

Remote afterloading devices used for high‐dose‐rate (HDR) brachytherapy may be supplied with different sources, and these sources typically have differing initial source strengths. In addition, the proposed frequency for source changes may also vary, depending upon the source type. Dosimetric parameters unique to each source are often used to compare source types. However, when considering the relative dosimetric efficiency between two HDR sources, the combined effect of source type, initial source strength, and source change scheme must be considered. A method of quantifying this combined effect by calculating mean dose rate from specific dosimetric source data is discussed. This method suggests an objective manner of comparing source scheme equivalency to facilitate performing a cost ratio analysis between different HDR sources and source change schemes.

PACS numbers: 87.53.Jw, 87.56.bg

## I. INTRODUCTION

Remote afterloading devices used for high‐dose‐rate brachytherapy are available with different source types and initial source strengths. Competing manufacturers currently offer different source activities combined with a variety of schedules for source change frequencies. When assessing the differences between source type and frequency of change, several subjective criteria related to performance and cost may need to be assessed. A method is proposed for objectively analyzing the relative dosimetric difference for combinations of isotope type, initial activity, and source change schedule by calculating mean dose rate at 1 cm on the transverse axis to compare treatment time and dosimetric efficiency.

## II. MATERIALS AND METHODS

When comparing remote afterloading devices used for HDR brachytherapy there are many issues to consider such as functionality, cost, regulatory matters, installation and shielding, availability of applicators and accessories, treatment planning, and dosimetry, to name a few. HDR remote afterloaders may be supplied with different source types and initial activities, and configured to have different source change schemes. While many of these considerations are somewhat subjective to evaluate, an objective method of evaluating the combined effect of isotope, initial activity, and source change schemes by the use of the concepts of integral activity^(1)^ and mean dose rate is proposed here.

The integral activity of a decaying radioactive source is a calculated value that represents the total number of disintegrations over a given time. The concept is especially useful when analyzing the effect of several source changes that occur at regular intervals with HDR after‐loading units. Typical HDR remote afterloading units use an ${}^{192}\text{Ir}$ source with a nominal source strength of about 370 GBq (10 Ci) changed at a frequency of every three months (microSelectron, Nucletron‐Elekta, Veenendaal, The Netherlands). An alternative HDR remote afterloader (MultiSource; Eckert & Ziegler BEBIG GmbH, Berlin, Germany) offers similar functionality; however, the isotope can be ${}^{60}\text{Co}$ with a nominal initial activity of 74 GBq (2 Ci) and the frequency for source change is proposed to be at every five years. A recent analysis by Palmer and Mzenda^(2)^ compared some of the dosimetric and economic aspects of these sources.

**Figure 1:**
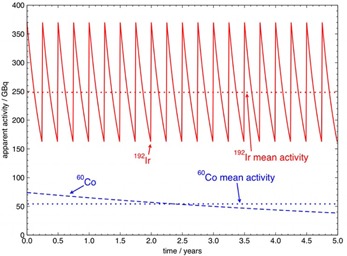
Proposed source activity and mean activity for a 370 GBq (10 Ci) HDR ${}^{192}\text{Ir}$ source replaced every three months and a 74 GBq (2 Ci) HDR ${}^{60}\text{Co}$ source replaced every five years, presented over a five‐year period.

Figure 1 shows the proposed source activity and mean activity over a five‐year period for the two different source schemes. The ${}^{192}\text{Ir}$ replacement schedule is based on a three‐month cycle and the initial activity at the time of each source replacement is anticipated to be 370 GBq. The nominal half‐life of ${}^{192}\text{Ir}$ is 73.83 days.^(3)^ The ${}^{60}\text{Co}$ replacement schedule is based on a five‐year cycle and the initial activity is 74 GBq. The nominal half‐life of ${}^{60}\text{Co}$ is 1,925 days.^(3)^ From Fig. 1 it can be seen that the effect of these two source schemes over time are quite different from each other and determining which, if either, of the two schemes is cost effective may be subjective.

## III. RESULTS & DISCUSSION

The concept of integral activity may be applied to represent the effect of the source change scheme and the source half‐life over a time period of interest.^(1)^ The integral activity of a decaying radioactive source, $A_{\text{INT}}$, from time zero to a given time, t, can be defined as the integration of the instantaneous activity, *A(t)*, as expressed in Eq. (1):

(1) $$A_{\text{INT}}=\int_{0}^{t}A(t^{\prime })dt^{\prime }$$

By substituting

(2) $$A(t)=A_{0}e^{-\lambda t}$$

where $\lambda =\text{ln}2/T_{1/2},\;T_{1/2}$ is the half‐life of the isotope, $A_{0}$ is the initial source activity, and *t* is the time of interest, a solution for $A_{\text{INT}}$ may be obtained as expressed in Eq. (3):

(3) $$A_{\text{INT}}=A_{0}\lambda^{-1}(1-e^{-\lambda t})$$

The quantity $A_{\text{INT}}$ has the dimensions of disintegrations. Dividing $A_{\text{INT}}$ by the time, *t*, expressed in seconds (Eq. (4)) yields the quantity of disintegrations per second, or becquerel (Bq). This quantity is numerically equal to a mean activity, *Ā*, over the time, t. In this case, *t* is the time during which the source being considered is installed and represents the interval for source changes (nominally three months for ${}^{192}\text{Ir}$ and five years for ${}^{60}\text{Co}$). A is expressed in Eq. (4):

(4) $$\bar{A}=A_{\text{INT}}/t$$

For the ${}^{192}\text{Ir}$ scheme of four source changes per year with an initial activity of 370 GBq, the following parameters may be defined: $A_{0}=3.7\times 10^{11}\;\text{Bq},\;T_{1/2}=73.83$ days, and t=91.31 days (one source change every three months). Substituting into Eq. (3), $A_{\text{INT}}=1.96\times 10^{18}$ disintegrations over a 91.31 day period. Using Eq. (4), a mean activity over the three‐month period of $2.48\times 10^{11}$ Bq (6.7 Ci) can be derived.

For the ${}^{60}\text{Co}$ scheme of one source change per five years with an initial activity of 74 GBq, the following parameters may be defined: $A_{0}=7.4\times 10^{10}\;\text{Bq},\;T_{1/2}=1925\;\text{days},\;\text{and}\;t=1826.25$ days (one source change every five years). Substituting into Eq. (3), $A_{\text{INT}}=8.56\times 10^{18}$ disintegrations over a five‐year period. Again using Eq. (4), a mean activity over a five‐year period of $5.42\times 10^{10}$ Bq (1.47 Ci) can be derived.

**Figure 2:**
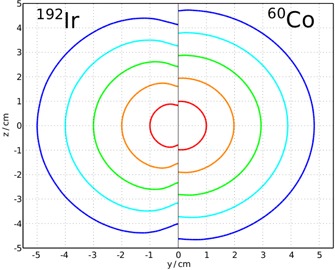
Isodose comparison of the HDR ${}^{192}\text{Ir}$ microSelectron v2^(6)^ and HDR ${}^{60}\text{Co}$ BEBIG Co0.A86^(7)^ sources. The Y direction is along the short axes of the sources and the Z direction is along the long axes. Arbitrary isodose lines are plotted, but the matching colors represent the same isodoses for both sources relative to the dose at 1 cm along the transverse axis.

In order to assess the treatment efficacy of these two source schedules, dose to a reference point according to the American Association of Physicists in Medicine (AAPM) Task Group No. 43 (TG‐43)^(^ ^4^ ^,^ ^5^ ^)^ formalism may also be considered. Using the TG‐43 formalism, the dose rate, *D*, to a point in water is determined using Eq. (5):

(5) $$\dot{D}(r,\theta )=S_{K}\cdot \Lambda \cdot \frac{G_{L}(r,\theta )}{G_{L}(r_{0},\theta_{0}})\cdot g_{L}(r)\cdot F(r,\theta )$$

where $S_{K}$ is the air‐kerma strength of the source, Λ is the dose‐rate constant, $G_{L}(r,\;\theta )$ is the geometry function, $g_{L}(r)$ is the radial dose function, and F(r,θ) is the 2D anisotropy function.^(5)^ The geometry function is independent of photon energy, and the radial dose function and the 2D anisotropy function are weakly dependent upon photon energy and are generally within ±10% when comparing ${}^{192}\text{Ir}$ to ${}^{60}\text{Co}$ over a range of 5 cm.^(^ ^6^ ^,^ ^7^ ^)^ An isodose comparison of the HDR ${}^{192}\text{Ir}$ microSelectron v2 and HDR ${}^{60}\text{Co}$ BEBIG Co0.A86 sources is presented in Fig. 2 for both sources normalized at 1 cm along the transverse axis. From Fig. 2 it may be seen that, for the purposes of this comparison, both sources have relatively similar dosimetric properties. Thus, in order to compare the relative efficiency of ${}^{192}\text{Ir}$ versus ${}^{60}\text{Co}$ for these two remote afterloading devices, the product of the air‐kerma strength and the dose‐rate constant alone may be used to calculate the dose rate $\left(Ḋ(r_{0},\theta_{0})\right)$ without respect to geometry, radial dose or anisotropy functions. Multiplying the mean activity by the air‐kerma rate constant $\Gamma_{\delta }(0.109\;\mu \text{Gy}\;m^{2}\;{\text{MBq}}^{-1}\;h^{-1}\;\text{for}\;{}^{192}\text{Ir}\;\text{and}\;0.309\;\mu \text{Gy}\;m^{2}\;{\text{MBq}}^{-1}\;h^{-1}\;\text{for}\;{}^{60}\text{Co})$ ^(8)^ yields the mean air‐kerma strength for each isotope, as shown in Eq. (6):

(6) $$\Gamma_{\delta }\cdot \bar{A}={\bar{S}}_{k}$$

When comparing HDR brachytherapy sources, the one with the best dosimetric efficiency is the one that delivers the largest dose over the proposed source change period. The mean dose rate, $\overset{―}{Ḋ(r_{0},\;\theta_{0})}$, can be used as a surrogate to assess dosimetric efficiency, and may be calculated using Eq. 7:

(7) $$\bar{\dot{D}(r_{0},\theta_{0})}={\bar{S}}_{K}\cdot \Lambda =\Gamma_{\delta }\cdot \bar{A}\cdot \Lambda =\Gamma_{\delta }\cdot \Lambda \cdot A_{0}{\left(\lambda t\right)}^{-1}(1-e^{-\lambda t})$$

For ${}^{192}\text{Ir}$, the mean dose rate, $\overset{―}{Ḋ(r_{0},\;\theta_{0})}$, for the proposed scheme of four source changes per year over five years can be calculated to be

$$1.09\times 10^{-7}\mu \text{Gy}\;m^{2}\;{\text{Bq}}^{-1}\;h^{-1}\cdot 2.48\times 10^{11}\;\text{Bq}\cdot 1.11\;\text{cGy}\;{\text{hr}}^{-1}\;U^{-1}=3.01\times 10^{4}\;\text{cGy}\;h^{-1}.$$

This can be compared to the ${}^{60}\text{Co}$ mean dose rate, $\overset{―}{Ḋ(r_{0},\;\theta_{0})}$, for the proposed scheme of one source change per five years of

$$3.09\times 10^{-7}\mu \text{Gy}\;m^{2}\;{\text{Bq}}^{-1}\;h^{-1}\cdot 5.42\times 10^{10}\;\text{Bq}\cdot 1.09\;\text{cGy}\;{\text{hr}}^{-1}\;U^{-1}=1.82\times 10^{4}\;\text{cGy}\;h^{-1}.$$

Data used to calculate the average dose rate are presented in Table 1.

**Figure 3:**
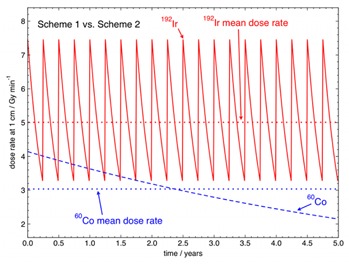
Instantaneous dose rates and mean dose rates at 1 cm in water in $\text{Gy}\;{\text{min}}^{-1}$ for ${}^{192}\text{Ir}$ for a 370 GBq (10 Ci) HDR ${}^{192}\text{Ir}$ source replaced every three months (Scheme 1) and a 74 GBq (2 Ci) HDR ${}^{60}\text{Co}$ source replaced every five years (Scheme 2), presented over a five‐year period.

Table 2 compares several initial source activities and source change schemes. Scheme 1 indicates the traditional source management scheme for ${}^{192}\text{Ir}$ afterloaders with four source changes per year. Scheme 2 lists the proposed source management scheme for a ${}^{60}\text{Co}$ afterloader with one source change every five years. Over a five‐year period it can be estimated that the ${}^{60}\text{Co}$ source with an initial activity of $7.40\times 10^{10}$ Bq (2 Ci) changed once every five years will, on average, be delivering a relative dose rate at 1 cm in water of $0.61\;(3.036\;\text{Gy}\;{\text{min}}^{-1}/5.010\;\text{Gy}\;{\text{min}}^{-1})$ as compared to an ${}^{192}\text{Ir}$ source with an initial activity of $3.70\times 10^{11}$ Bq (10 Ci) changed four times per year over the same five‐year period. Thus the ${}^{60}\text{Co}$ scheme of one source change per five years will be 61% as efficient as the ${}^{192}\text{Ir}$ source scheme of four changes per year over the same five‐year period in terms of treatment time delivery. Figure 3 shows the actual dose rate and the mean dose rate at 1 cm in water in $\text{Gy}\;{\text{min}}^{-1}$ for the ${}^{192}\text{Ir}$ source change Scheme 1 and the ${}^{60}\text{Co}$ source change Scheme 2 over a five‐year period.

**Table 1:** Constants related to ${}^{192}\text{Ir}$ and ${}^{60}\text{Co}$ HDR sources

		*Isotope*	
*Quantity*	*Unit*	${}^{192}\text{Ir}$	${}^{60}\text{Co}$
Half‐life	days	73.83^(3)^	1925^(3)^
Mean photon energy	MeV	0.37^(6)^	1.25^(3)^
Air‐kerma rate constant	$\mu \text{Gy}\;m^{2}\;{\text{Bq}}^{-1}\;h^{-1}$	$1.091\times 10^{-7(8)}$	$3.090\times 10^{-7(8)}$
Dose‐rate constant	$\text{cGy}\;h^{-1}\;U^{-1}$	1.109^(6)^	1.087^(7)^

**Table 2:** ${}^{192}\text{Ir}$ conventional source change scheme (Scheme 1) compared to proposed (Scheme 2) and theoretical (Schemes 3–5) source change schemes for ${}^{60}\text{Co}.$

	*Source Change Scheme and Isotope*				
	*Scheme 1*	*Scheme 2*	*Scheme 3*	*Scheme 4*	*Scheme 5*
*Quantity*	${}^{192}\text{Ir}$	${}^{60}\text{Co}$	${}^{60}\text{Co}$	${}^{60}\text{Co}$	${}^{60}\text{Co}$
Initial activity (Bq)	$3.70\times 10^{11}$	$7.4\times 10^{10}$	$1.221\times 10^{11}$	$1.084\times 10^{11}$	$9.102\times 10^{10}$
Initial activity (Ci)	10	2	3.30	2.93	2.46
Source change time (years)	0.25	5	5	3	0.25
Integrated activity (disintegrations)	$1.960\times 10^{18}$	$8.557\times 10^{18}$	$1.412\times 10^{19}$	$8.481\times 10^{18}$	$7.064\times 10^{17}$
Mean activity (Bq)	$2.485\times 10^{11}$	$5.423\times 10^{10}$	$8.948\times 10^{10}$	$8.958\times 10^{10}$	$8.954\times 10^{10}$
Mean activity (Ci)	6.715	1.466	2.418	2.421	2.420
Mean air‐kerma strength $\left(\mu \text{Gy}\;m^{2}\;h^{-1}\right)$	$2.711\times 10^{4}$	$1.676\times 10^{4}$	$2.765\times 10^{4}$	$2.768\times 10^{4}$	$2.767\times 10^{4}$
Mean dose rate at 1 cm in water $\left(\text{cGy}h^{-1}\right)$	$3.006\times 10^{4}$	$1.821\times 10^{4}$	$3.005\times 10^{4}$	$3.009\times 10^{4}$	$3.007\times 10^{4}$
Mean dose rate at 1 cm in water $\left(\text{Gy}\;{\text{min}}^{-1}\right)$	5.010	3.036	5.009	5.015	5.012

A relational comparison may also be used to establish equivalent source change schemes by relating Eq. (7) for both isotopes, and solving for either $A_{0,\text{Co}-60}$ (the initial Cobalt activity) or $t_{\text{Co}-60}$ (the proposed time between ${}^{60}\text{Co}$ source changes) or both.

**Figure 4:**
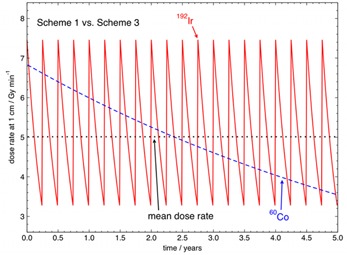
Instantaneous dose rates and mean dose rate at 1 cm in water in $\text{Gy}\;{\text{min}}^{-1}$ for ${}^{192}\text{Ir}$ for a 370 GBq (10 Ci) HDR ${}^{192}\text{Ir}$ source replaced every three months (Scheme 1) and a 122 GBq (3.30 Ci) HDR ${}^{60}\text{Co}$ source replaced every five years (Scheme 3), presented over a five‐year period.

For example, a ${}^{60}\text{Co}$ source scheme with a theoretical initial activity, $A_{0,\text{Co}-60}$, of 122 GBq (3.30 Ci) with a source change every five years, (Scheme 3 in Table 2) would have a numerical mean dose rate equivalence to that of ${}^{192}\text{Ir}$ with $A_{0,\text{Ir}-192}$ of 370 GBq changed every three months over a five‐year period. One might expect the higher ${}^{60}\text{Co}$ source activity in Scheme 3 to incur a higher cost than that of ${}^{60}\text{Co}$ in Scheme 2, and the source may need to be increased in diameter or length due to specific activity limitations. Other possible factors related to the higher activity, such as a consideration for extra shielding of the afterloader and/or the treatment room bunker, might also add to the cost. Figure 4 shows the actual dose rate and the mean dose rate at 1 cm in water in $\text{Gy}\;{\text{min}}^{-1}$ for ${}^{192}\text{Ir}$ for source change Scheme 1 and for ${}^{60}\text{Co}$ for source change Scheme 3 over a five‐year period. For this arrangement for ${}^{60}\text{Co}$ (Scheme 3), it can be seen that the mean dose rates have become equal over the five‐year period.

**Figure 5:**
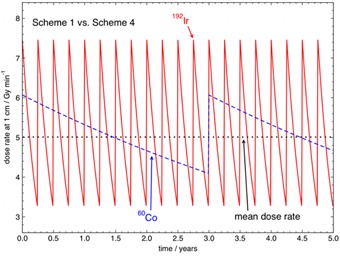
Instantaneous dose rates and mean dose rate at 1 cm in water in $\text{Gy}\;{\text{min}}^{-1}$ for ${}^{192}\text{Ir}$ for a 370 GBq (10 Ci) HDR ${}^{192}\text{Ir}$ source replaced every three months (Scheme 1) and a 108 GBq (2.93 Ci) HDR ${}^{60}\text{Co}$ source replaced every three years (Scheme 4), presented over a five‐year period.

Alternatively, a ${}^{60}\text{Co}$ source scheme with a theoretical initial activity, $A_{0,\text{Co}-60}$, of 108 GBq (2.93 Ci) and a source change every three years (Scheme 4 in Table 2) would also satisfy an ${}^{192}\text{Ir}$ mean dose rate equivalence with an $A_{0,\text{Ir}-192}$ of 370 GBq (10 Ci) changed every three months. In this scheme, the higher source activity and more frequent source change schedule might incur a significantly higher cost than the traditional ${}^{192}\text{Ir}$ schedule of Scheme 1, or the two ${}^{60}\text{Co}$ schemes (Scheme 2 and 3). Again, the potential need for extra shielding of the after‐loader and possibly the treatment room bunker may need to be accounted for. Figure 5 shows a comparison between the ${}^{192}\text{Ir}$ source change Scheme 1 and the ${}^{60}\text{Co}$ source change Scheme 4 over a five‐year period. Schemes 1 and 4 deliver equivalent mean dose rates.

**Figure 6:**
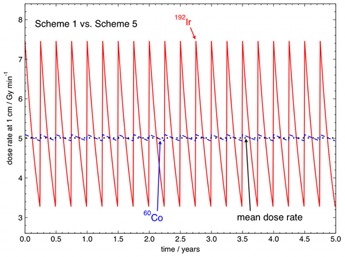
Instantaneous dose rates and mean dose rates at 1 cm in water in $\text{Gy}\;{\text{min}}^{-1}$ for ${}^{192}\text{Ir}$ for a 370 GBq (10 Ci) HDR ${}^{192}\text{Ir}$ source replaced every three months (Scheme 1) and a 91.0 GBq (2.46 Ci) HDR ${}^{60}\text{Co}$ source replaced every three months (Scheme 2), presented over a five‐year period.

The proposed initial activity of ${}^{60}\text{Co}\;(A_{0,\text{Co}-60})$ of 74 GBq (2.00 Ci) can never be equivalent in dosimetric terms to the ${}^{192}\text{Ir}$ with $A_{0,\text{Ir}-192}$ of370 GBq changed every three months. However, an $A_{0,\text{Co}-60}$ of 91.0 GBq (2.46 Ci) changed every three months (Scheme 5 in Table 2) will be dosimetrically equivalent to Scheme 1, although this scheme would likely have little practical benefit over the traditional ${}^{192}\text{Ir}$ source change scheme. Figure 6 shows a comparison between the ${}^{192}\text{Ir}$ source change Scheme 1 and the ${}^{60}\text{Co}$ source change Scheme 5 over a five‐year period. Schemes 1 and 5 deliver equivalent mean dose rates.

The effect of a somewhat different treatment time averaged over a number of years may be judged to be clinically significant or not; however, this method suggests a formalism to objectively assess the relative efficiency of different source management schemes for isotopes such as ${}^{192}\text{Ir}$ and ${}^{60}\text{Co}$ that have similar brachytherapy dose characteristics (as suggested by Fig. 2). Source change Schemes 1 to 5 are summarized in Table 2.

Some of the operating costs associated with running an HDR suite may be associated with the replacement cost of the source (S), the yearly service costs (M), the number of source changes per year (*n*), and the amortization period (*a*) over which the cost is determined. Other fixed operating costs including personnel, patient applicators and expendables, facility costs and financial servicing costs are unrelated to the source type and source change scheme. An approximation of the source related costs (*C*) over the amortization period (a) may be estimated in Eq. 8 to be:

(8) C=((n⋅S)+M)⋅a

Equation (8) is somewhat simplistic as it neglects the differences in dosimetric efficiency discussed above. In order to make a fairer cost ratio analysis between competing schemes, it might be useful to also account for these differences in relative dosimetric efficiency. If for example, as in Scheme 1, a source change for ${}^{192}\text{Ir}$ at four changes per year costs *S* per source, then, based upon the source efficiency alone, one might expect the cost for Scheme 2 ${}^{60}\text{Co}$ source change every five years to be less than or equal to 0.61×S to account for the relative dosimetric inefficiency of ${}^{60}\text{Co}$ (Scheme 2), as compared to ${}^{192}\text{Ir}$ (Scheme 1). Other factors, such as differences in servicing costs and the amortization period, would also need to be considered, as suggested in Eq. (8). Schemes 3, 4, and 5 attempt to balance out the dosimetric efficiency by using theoretical higher initial source activities, but come at the potential increased cost of higher initial source activities, more frequent source changes, or shorter amortization periods. Since Schemes 1, 3, 4, and 5 have the same dosimetric efficiency, Eq. (8) can be used directly to compare the source related costs. It is possible that any of the ${}^{60}\text{Co}$ source change Schemes (2, 3, 4, or 5) in Table 2 may prove to be cost effective when compared to the traditional ${}^{192}\text{Ir}$ source change (Scheme 1) in Table 2. However, an objective assessment of the relative dosimetric efficiency may also prove to be of interest when incorporated within a cost ratio analysis.

## IV. CONCLUSIONS

Many factors need to be considered when choosing between different remote HDR afterloading units including functionality, cost, regulatory matters, installation and shielding, availability of applicators and accessories, treatment planning, and dosimetry, to name a few. Changing to a different manufacturer may mean a complete refresh of applicators and accessories, and this may incur a heavy financial penalty. Manufacturers providing different source types may also have unique considerations for radiation safety in terms of bunker shielding and device shielding. Many of these considerations are difficult to quantify and must be addressed with some care.

On the other hand, it is possible to make a comparative assessment of the relative dosimetric efficiency of different isotopes and proposed source change schemes. We present here a method whereby mean dose rate may be used for quantifying the relative dosimetric efficiency between ${}^{192}\text{Ir}$ and ${}^{60}\text{Co}$ sources by quantifying different source change schemes. This method suggests an objective manner of comparing source scheme equivalency when performing a cost ratio analysis between different HDR sources and source change schemes.
